# Supplementary material for: Passive directional sub-ambient daytime radiative cooling
Source: Nat Commun. 2018 Nov 27;9:5001. doi: 10.1038/s41467-018-07293-9 (PMC6258698; doi:10.1038/s41467-018-07293-9)
Supplement: Supplementary file 1 — Supplementary Information [file 41467_2018_7293_MOESM1_ESM.pdf]

Supplementary Information

Passive directional sub-ambient daytime radiative cooling

Bikram Bhatia[1*], Arny Leroy[1*], Yichen Shen[2*], Lin Zhao[1], Melissa Gianello[1], Duanhui Li[3], Tian Gu[3], Juejun Hu[3], Marin Soljačić[2], and Evelyn N. Wang[1]

[1]Department of Mechanical Engineering, Massachusetts Institute of Technology, Cambridge, Massachusetts 02139, USA
[2]Department of Physics, Massachusetts Institute of Technology, Cambridge, Massachusetts 02139, USA
[3]Department of Materials Science and Engineering, Massachusetts Institute of Technology, Cambridge, Massachusetts 02139, USA

[*]emails: bikram@mit.edu (B.B.), aleroy@mit.edu (A.L.), ycshen@mit.edu (Y.S.)

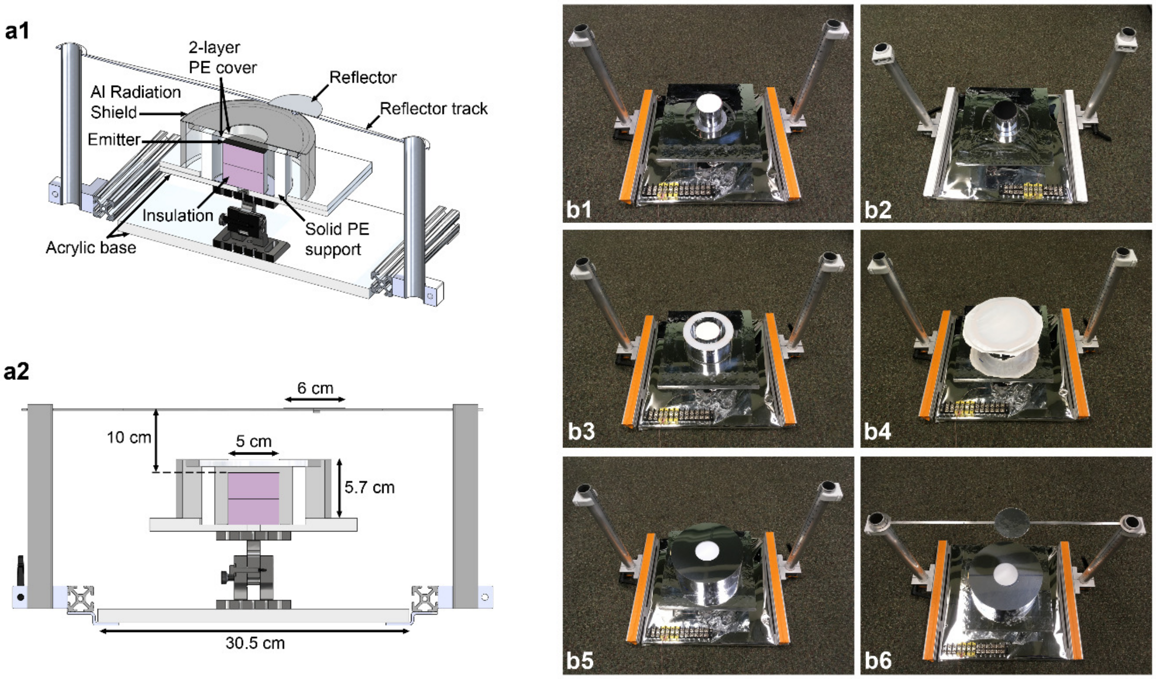

Supplementary Figure 1. Device construction. Device cross-section trimetric (**a1**) and front view (**a2**). Images showing different device components: solar-white (**b1**) and solar-black (**b2**) emitters placed over thermal insulation, solid polyethylene (PE) support (**b3**), 2-layer polyethylene cover (**b4**), polished aluminum radiation shield and aperture (**b5**), and direct-solar reflector (**b6**). (Details in Supplementary Note 2).

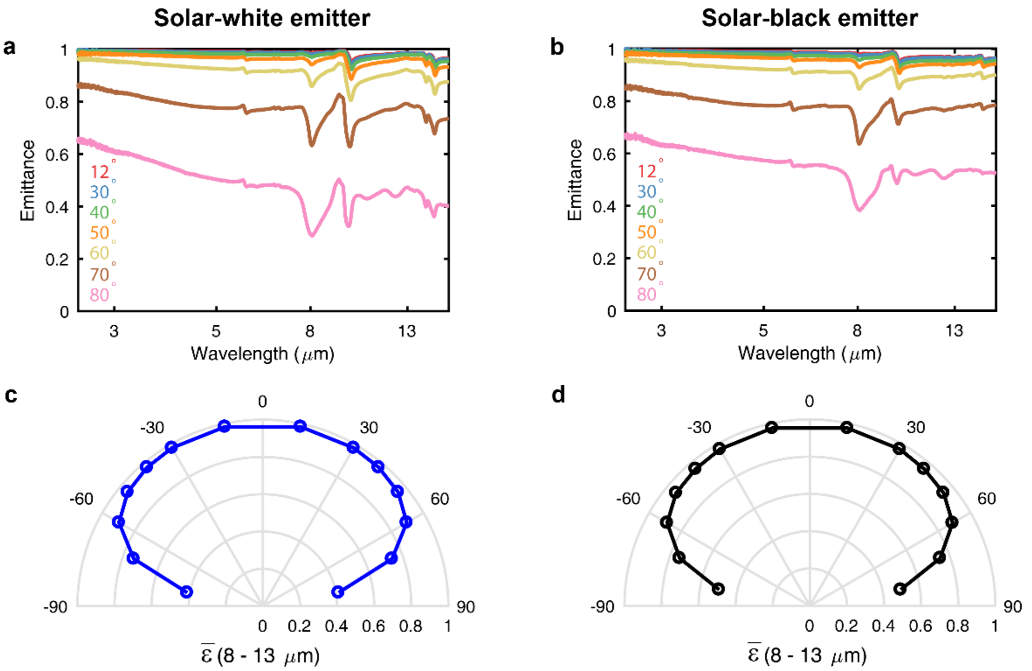

Supplementary Figure 2. Measured wavelength- and angle-dependent emissivity. Measured spectral emissivity of the solar-white emitter (**a**) and solar-black emitter (**b**) at different angles of incidence. Average emissivity in the high-transparency atmospheric spectral window (8-13 μm) plotted as a function of incidence angle (**c**: solar-white emitter, **d**: solar-black emitter). (Details in Supplementary Note 3).

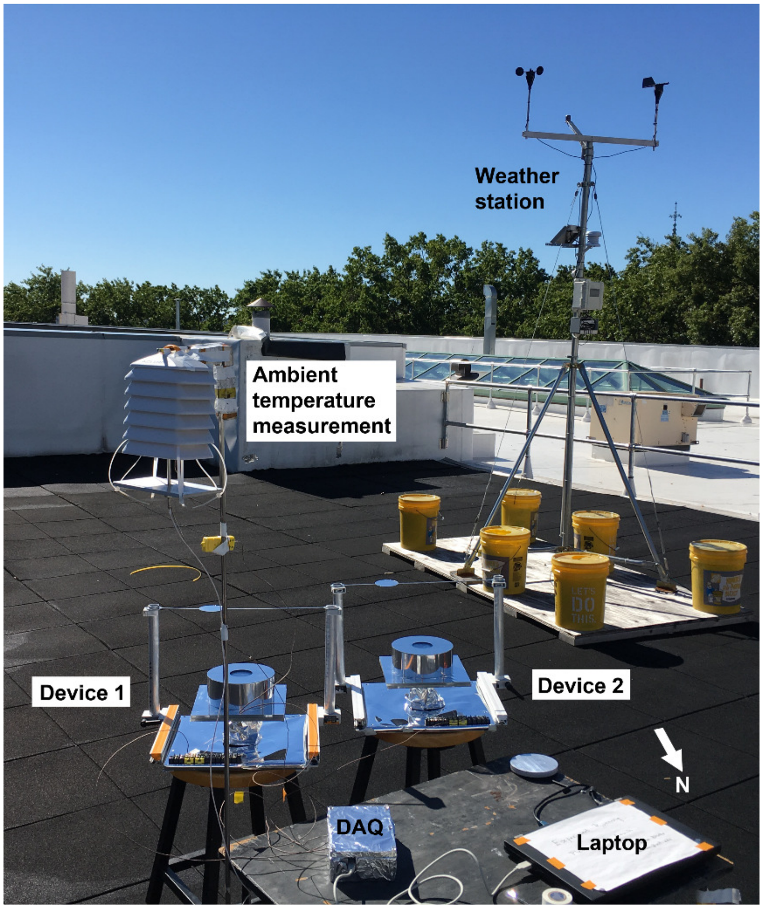

Supplementary Figure 3. Measurement setup. Image of the rooftop measurement setup showing the devices, data acquisition and weather monitoring equipment. (Details in Supplementary Note 4).

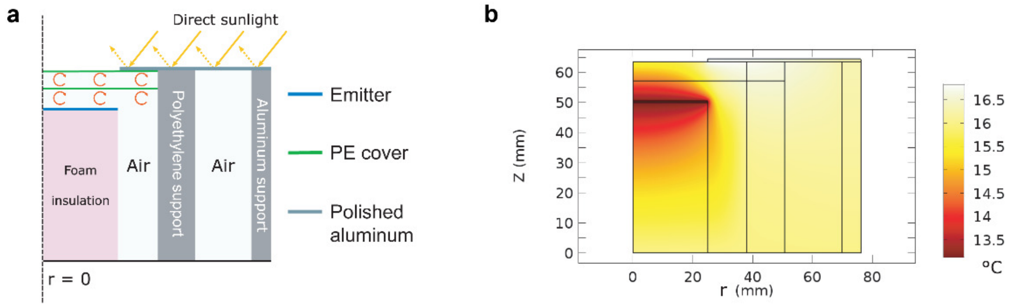

Supplementary Figure 4. Theoretical simulation of the temperature distribution of the device. a, Conjugate conduction and natural convection heat transfer model. **b**, Steady-state temperature distribution shown for half of the device cross-section. The emitter cooling power is 20 Wm^{-2} and the ambient temperature is 16 °C. (Details in Supplementary Note 5).

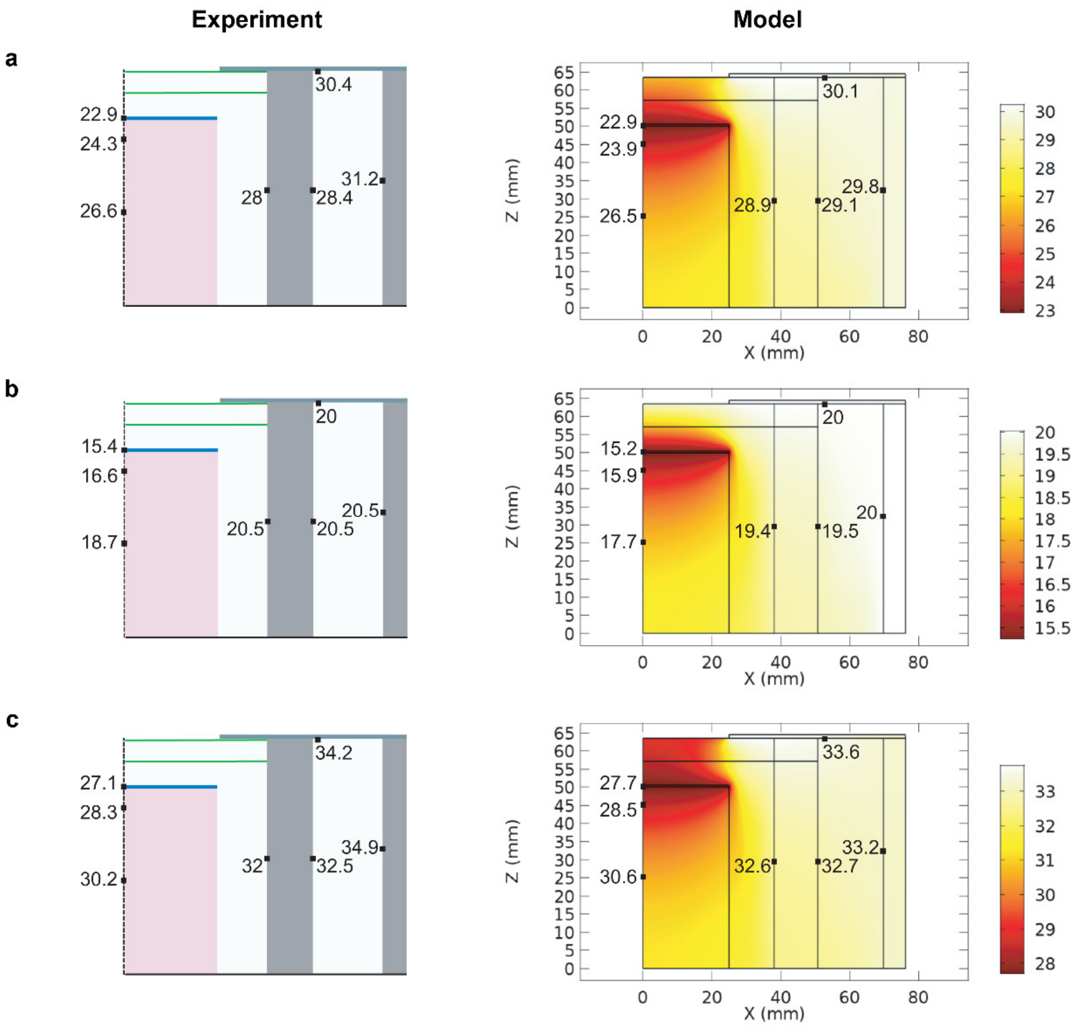

Supplementary Figure 5. Model validation. Comparison of the experimentally measured temperatures at different locations of the device (left) with the temperature distribution predicted using the COMSOL model (right). Results are shown for three different conditions: **a**, 1.5 hours after solar noon on a mostly clear day with scattered clouds (average ambient temperature: 26.8 °C), **b**, clear night (average ambient temperature: 20.1 °C), and **c**, around solar noon on a hazy day with some clouds (average ambient temperature: 29.0 °C). Measurements were done on the rooftop in Cambridge, MA between 2 pm August 23, 2018 and 2 pm August 24, 2018. (Details in Supplementary Note 5).

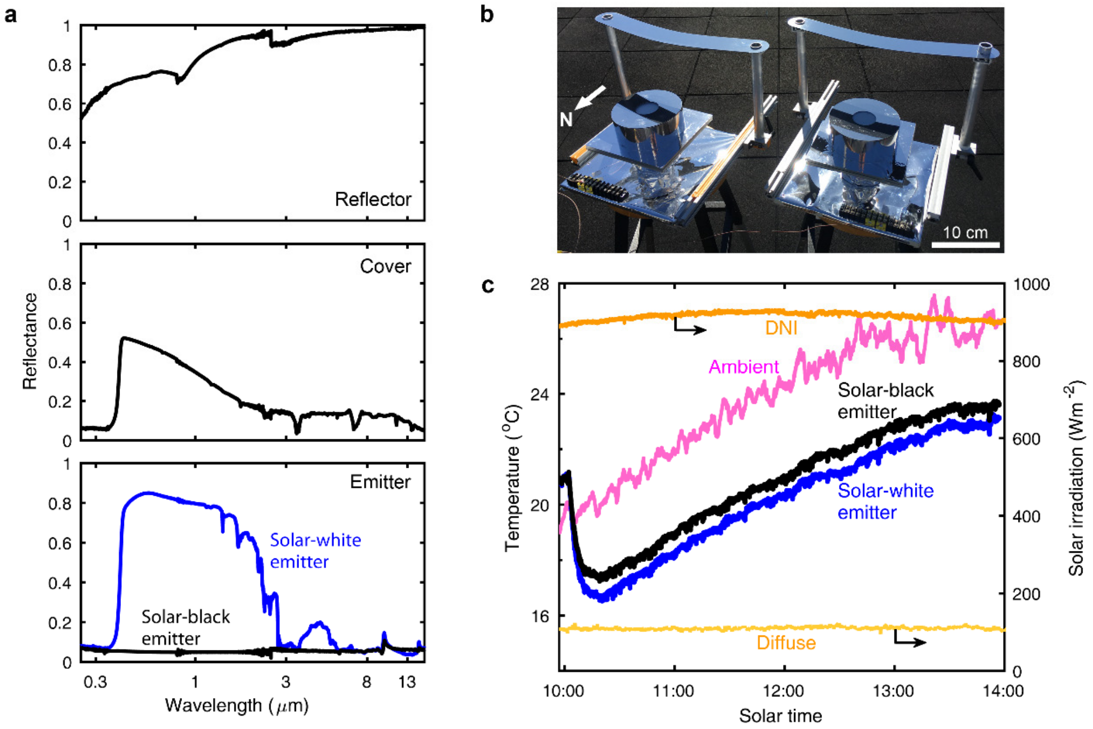

Supplementary Figure 6. Stagnation temperature measurement using a non-solar-tracking setup. a, Spectral direct-hemispherical reflectance of the polished aluminum fixed reflector, white polyethylene (from a grocery bag) cover and white- and black-painted emitters. **b**, A photograph of the two devices during measurement. **c**, Temperature of the solar-white and solar-black emitters measured two hours before and two hours after solar noon. Measured ambient temperature and direct normal irradiance (DNI) and diffuse solar irradiation are also shown for reference. The measurement was done on a clear day in Cambridge, MA on October 4, 2017. (Details in Supplementary Note 6).

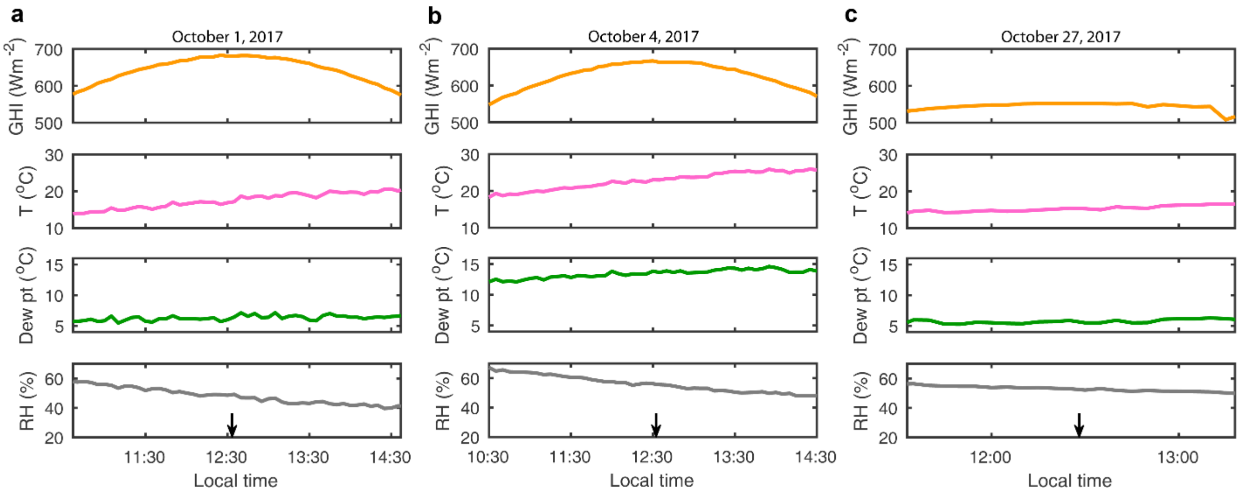

Supplementary Figure 7. Weather parameters. Global horizontal irradiance, ambient temperature, dew point and relative humidity measured during the course of measurements shown in Figure 3 (**a**), Supplementary Figure 6 (**b**) and Figure 4 (**c**). The x-axis shows the local time and the downward pointing arrow represents solar noon. Measurement location: Cambridge, MA. (Details in Supplementary Note 7).

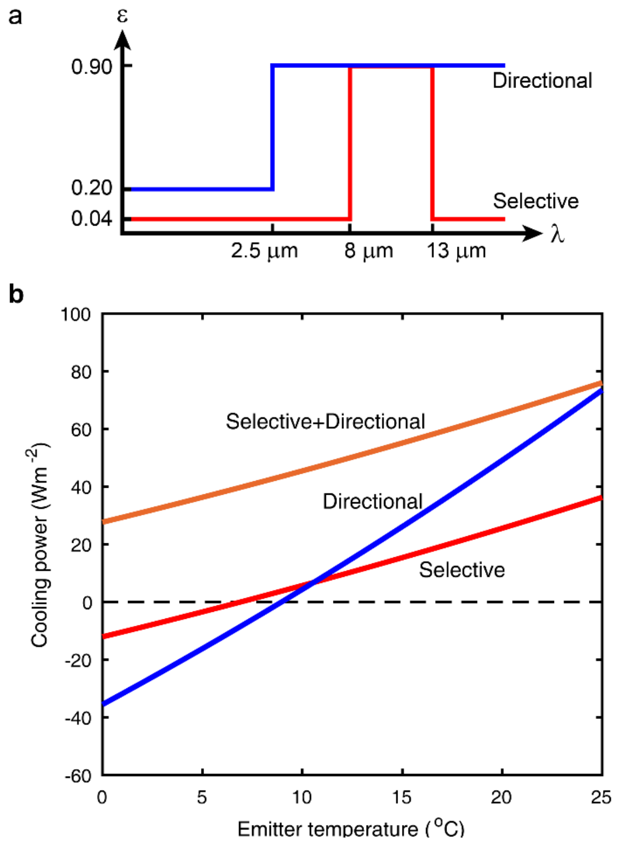

Supplementary Figure 8. Cooling performance comparison. a, Emitter spectral properties used to compare the daytime radiative cooling using selective and directional approaches. **b**, Modeled cooling power as a function of emitter temperature (ambient temperature: 25 °C) during the day for a solar-reflecting square-wave-type Selective emitter, partly solar-reflecting step-function-type emitter for the Directional approach, and the square-wave-type selective emitter coupled with the directional approach to reject direct sunlight for the Selective+Directional case. (Details in Supplementary Note 8).

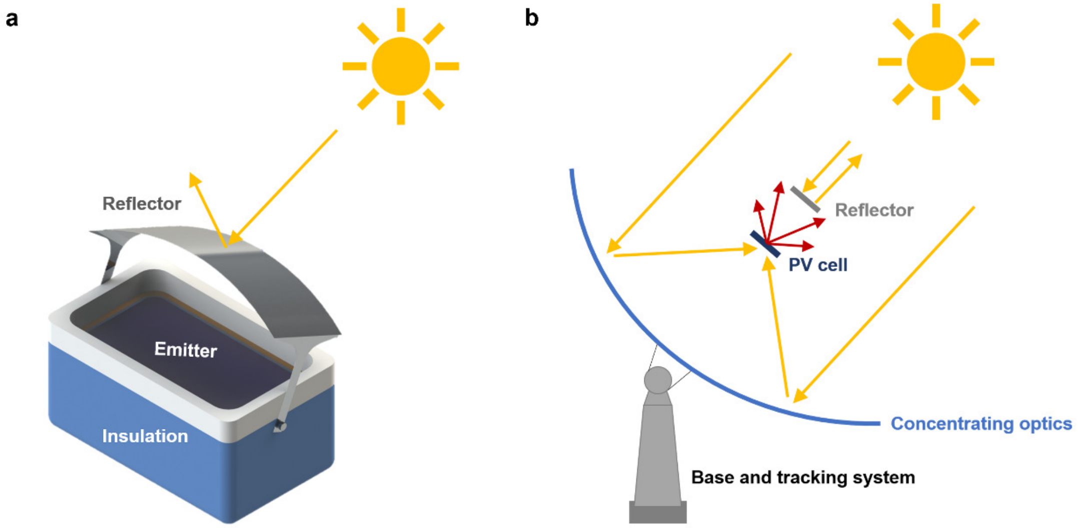

Supplementary Figure 9. Possible applications. Concept drawings showing possible approaches for practical implementation of directional daytime radiative cooling in portable refrigeration (**a**) and concentrated photovoltaic systems (**b**). (Details in Supplementary Note 9).

Supplementary Note 1: Diffuse radiation modeling

The total solar radiation incident on a surface can be classified into its diffuse and direct beam components. The direct beam component from the solar disk was completely reflected in our experiment. The diffuse component accounts for the solar radiation contribution from the sky outside the solar disk. We estimated the diffuse fraction (I_d) of the total solar radiation (I) using the Erbs et al. correlation[1]:

$$\frac{I_d}{I} = \begin{cases} 1.0 - 0.09k_T & \text{for } k_T \le 0.22 \\ 0.9511 - 0.1604k_T + 4.388k_T^2 - 16.638k_T^3 + 12.336k_T^4 & \text{for } 0.22 < k_T \le 0.80 \\ 0.165 & \text{for } k_T > 0.8 \end{cases} \tag{1}$$

where $k_T = \frac{I}{I_o}$ is the clearness defined using the total global radiation, I, calculated from the AM1.5 solar spectrum and the total extraterrestrial radiation, I_o, calculated from the AM0 solar spectrum. The direct beam radiation, I_b, is thus simply equal to $I - I_d$. The diffuse contribution can be further classified into (1) the isotropic contribution received uniformly across the entire sky dome, (2) the circumsolar contribution from the region around the solar disk, and (3) the horizon brightening contribution concentrated near the horizon. For our experiment, comprising a horizontal surface without optical access to the horizon and the region around the sun blocked by a reflector, we are able to neglect the circumsolar contribution and horizon brightening and treat the diffuse solar radiation as uniform across the sky. The isotropic diffuse radiation, $I_{d,iso}$, for a horizontal surface is estimated using the HDKR model[1]:

$$I_{d,iso} = I_d\left(1 - A_i\right), \text{ where } A_i = \frac{I_b}{I_o}. \tag{2}$$

Supplementary Equations 1 and 2 were used to calculate the isotropic diffuse spectral irradiance $I_{\mathrm{d,iso}}(\lambda)$ (units: $Wm^{-2}\mu m^{-1}$) assuming the same spectral distribution for the diffuse and direct beam components.[1] The diffuse solar spectral radiance (units: $Wm^{-2}\mu m^{-1}sr^{-1}$), $I_{\mathrm{solar\text{-}diffuse}}(\lambda)$, used in Equation 4 of the main text, was calculated by dividing $I_{\mathrm{d,iso}}(\lambda)$ by the solid angle of the integration domain.

Supplementary Note 2: Device design and fabrication

Supplementary Figure 1 shows the cross-section CAD drawings and photographs of the fabricated device assembly. The device consisted of a disk-shaped copper emitter, 5 cm in diameter and 0.5 mm thick. The top side of the emitter was painted using three coats of flat white or flat black spray paint (Krylon Colormaster®) that were relatively black in the mid-infrared wavelengths. The emitter rested on two layers of 2.5 cm thick extruded polystyrene thermal insulation (FOAMULAR® 150) cut to match the diameter of the emitter. The insulation was surrounded by a solid polyethylene (PE) tube (inner diameter: 7.6 cm, outer diameter: 10.2 cm), which served as support for the convection cover. The diffuse-solar-reflecting convection cover was made using two 16 μm thick sheets of nanoporous polyethylene (Targray Technology International Inc., PE Separator Wet-Stretch) attached to a 6.4 mm thick aluminum ring (inner diameter: 10.7 cm, outer diameter 12.7 cm). This assembly was covered with a 5.7 cm tall polished aluminum hollow cylinder (inner diameter: 14 cm, outer diameter: 15.2 cm) with a polished aluminum sheet on top containing a 5 cm diameter aperture for the emitter. The device assembly was mounted on an acrylic base. The curved surfaces of the thermal insulation and solid PE support, as well as the acrylic base were covered with aluminized Mylar to minimize radiative transfer and solar absorption. The reflector assembly was mounted to the acrylic base using 80/20 framing that allowed the hollow rods supporting the reflector track to move relative to the emitter. The reflector comprised of a 6 cm diameter polished aluminum disk capable of moving along a custom-fabricated (using water jet) aluminum track. The height of the reflector was fixed at approximately 15 cm above the emitter.

Supplementary Note 3: Spectral-Angular emissivity of the emitters

Supplementary Figure 2 shows the measured spectral and angular emissivity of the solar-white and solar-black emitters used for the experiments. The angle-dependent emissivity, averaged over s- and p-polarizations, was evaluated from reflectance measurements done using an FTIR spectrometer (Nicolet 6700, Thermo Scientific) with variable angle accessories (SpectraTech variable angle accessory and Harrick Scientific's 12º incident angle Specular Reflection Accessory). The spectral measurement results shown in Supplementary Figures 2a and 2b are consistent with the direct-hemispherical reflectance shown in Figure 2c in the main text. The measured emissivity is nearly constant for all angles relevant for radiative transfer within the experimental setup (around normal direction due to the small spacing between the emitter, diffuse-solar-reflecting cover and aluminum aperture).

Supplementary Note 4: Measurement setup

Supplementary Figure 3 shows an image of the measurement setup used for outdoor measurements. The setup comprised of two devices, each consisting of a thin copper emitter attached with thermocouples (and Kapton heaters connected to a source meter, for the cooling power measurement experiment – Figure 4 of the main text) on the bottom side. Temperature data was acquired using a DAQ module (Measurement Computing USB-TC) connected to a laptop. The DAQ device was enclosed in an aluminum box covered with aluminum foil to minimize heating due to direct sunlight and maintain a relatively isothermal environment. The ambient temperature was measured using an exposed element RTD (Omega P-L-A-1/4-6-1/4-T-6) designed for accurate air temperature measurement. The RTD was suspended ~5 ft. above the ground inside a solar radiation shield that prevented heating due to solar radiation while allowing air flow. Supplementary Figure 3 also shows the weather station in the background that was used for weather monitoring during the course of the experiment (refer Supplementary Note 7 for more details). A separate pyrheliometer and pyranometer assembly mounted on a high-precision 2-axis solar-tracker was also installed on the rooftop (not shown in Supplementary Figure 3), with the two sensors always aligned towards the sun. These sensors were used to measure the direct normal irradiance (DNI) and global tilted irradiance (GTI).

Supplementary Note 5: Device COMSOL modeling

We developed a theoretical model using COMSOL to simulate heat transfer and estimate the temperature distribution within the device. The model is shown in Supplementary Figure 4a, where the geometry of each component matches the real device. A conjugate conduction and natural convection heat transfer model was used to capture both conduction in solid materials and natural convection in air gaps. The heating effect of the direct sunlight incident on the aluminum cover was included by using the solar absorption of the polished aluminum (0.2). Other external boundary conditions were defined using convection correlations with respect to the ambient temperature – we used a constant wind speed around 7.6 ms^{-1} based on the conditions during the experiment. Heat conduction loss through heater wires was also estimated and included in the heat transfer coefficient calculation. An example of the simulated steady-state temperature distribution of the device is shown in Supplementary Figure 4b, when the emitter cooling power is 20 Wm^{-2} and the ambient temperature is 16 °C. The predicted steady-state emitter temperature is 13 °C, which matches our experimental results under similar conditions (Figure 4b main text).

In order to experimentally validate the temperature distribution predicted using the COMSOL model, we installed six additional thermocouples at different locations within the device with the solar-white emitter. We then performed three outdoor measurements (each 1 hour long), following a procedure similar to that used to obtain results shown in Figure 3, under different solar irradiation and weather conditions. Supplementary Figure 5 shows the experimentally measured average temperatures for each of the three experiments (averaged after reaching thermal equilibrium). Supplementary Figure 5 also shows the temperature distribution and temperatures evaluated at the thermocouple locations using the COMSOL model with the corresponding experimental conditions as the input. The modeled temperature distribution within the device shows good

agreement with experimental results under different conditions. Minor deviations between experiments and model results can be attributed to thermocouple accuracy, variability in positioning the thermocouples and optical properties of different surfaces. Overall, the agreement between experimental and modeling results validates the COMSOL model used to estimate the conductive-convective heat transfer coefficient.

Supplementary Note 6: Non-tracking, low density polyethylene experiment

We modified the device configuration to demonstrate the possibility of sub-ambient passive cooling without solar tracking (Supplementary Figure 6). We replaced the disk-type reflector (60 mm diameter) that required adjustment with changing sun position (Figure 2) with a band-type direct-solar reflector of the same width as the disk-reflector diameter. The shape of the band reflector was determined using the solar-reflector tracking algorithm utilized to calculate the track path for the disk-type solar reflector (described in the Methods section). In addition, to demonstrate the possibility of achieving sub-ambient daytime cooling using common household materials, we replaced the 2-layer nanoporous polyethylene cover with a cover made using two layers of white low-density polyethylene (LDPE, each ~50 μm thick) taken from a grocery bag. Supplementary Figure 6a shows the spectral reflectance of the double-layer LDPE convection cover – the solar-weighted reflectance was 39% and an average transmittance was 67% in the atmospheric window, in comparison with double-layer nanoporous polyethylene with 55% solar reflectance and 92% atmospheric-window transmittance. The rest of the setup, including the solar-white and solar-black emitters, was the same as shown in Figure 2 of the main text.

To demonstrate the cooling performance of the modified setup with the band reflector and white LDPE cover grocery bag, we performed a stagnation temperature measurement around solar noon using the same procedure discussed in Figure 3. Supplementary Figure 6c shows the results of the stagnation temperature measurement. The average reduction of the device stagnation temperature was ≈4 °C below the ambient temperature and the solar-white emitter was cooler than the solar-black emitter by ≈0.4 °C. The measured stagnation temperature reduction using the modified setup was comparable to the ≈5 °C cooling achieved using the setup used in Figure 2. The slight reduction in performance can be partly attributed to the lower solar reflectance and lower

atmospheric-window transmittance of the LDPE cover which increased the contribution of the diffuse solar radiation and reduced the net outgoing mid-IR radiation. Further reduction in the cooling power was due to the larger solid angle subtended by the band-type direct-solar reflector on the emitter (as compared to the disk-type reflector) which reduced the angular domain available for mid-IR emission and increased the radiation emitted by the reflector towards the emitter. Overall the significant reduction of device temperature below ambient temperature even with this sub-optimal setup made using readily available household materials demonstrates the ease of implementation and potential of this approach.

Supplementary Note 7: Weather data for all measurements

A weather station (HOBO U30 Weather Station) installed on the rooftop (same location as the experimental setup) was used for weather monitoring. The weather station measured the global horizontal irradiance (GHI, using a pyranometer sensor), ambient air temperature, dew point and relative humidity. The data acquisition frequency was set at 5 minutes. Supplementary Figure 7 shows the measured weather parameters during the course of measurements reported in Figures 3 and 4, and Supplementary Figure 6.

Supplementary Note 8: Directional daytime radiative cooling – Potential

This section explores the potential benefits of the directional approach to passive daytime radiative cooling, compares its performance with existing method using spectrally selective surfaces, and demonstrates the possibility of combining the two to achieve significantly improved performance. To do so, we evaluated the cooling performance (using the modeling framework shown in the main text) for representative spectrally selective and directional configurations. Supplementary Figure 8a shows the spectral emissivity of the emitters chosen for this analysis. For the "Spectral" approach, we used a square-wave-type spectrally selective emitter with an emissivity of 0.90 in the transparent atmospheric spectral window and a reflectivity of 0.96 (emissivity of 0.04) at all other wavelengths, including in the solar spectrum – representative of existing literature on spectrally selective surfaces for daytime radiative cooling.[2–4] For the "Directional" approach, we used a step-function-type partly-solar-reflecting emitter with a reflectivity of 0.80 (emissivity of 0.20) in the solar spectrum (<2.5 μm) and an emissivity of 0.90 at longer wavelengths, including in the transparent atmospheric window – similar to the solar-white emitter used in the current study. In addition, we assumed a direct-solar reflector with a reflectivity of 0.96 at all wavelengths. No diffuse-solar-reflecting cover was considered for the ease of comparison.

Supplementary Figure 8b compares the cooling performance of the different cases, assuming only radiative contributions (*i.e.*, $h_{\text{cond-conv}} = 0$). We observe that while the Selective approach results in a lower emitter temperature, the Directional approach can achieve a significantly higher maximum cooling power than the Selective approach. The higher cooling power is primarily a result of how the direct solar radiation is rejected in the two cases. In the Selective case, the 0.96 solar reflectance translates to a direct reduction of cooling power during the day by ~40 Wm^{-2}. On the other hand, there is practically no cooling power penalty from the direct solar radiation in the Directional case.

In addition, because of the confinement of the solar disk in the sky, the angular window blocked in the Directional approach to reject direct sunlight is significantly smaller than the spectral window blocked by square-wave-type emitters in the Selective approach resulting in a higher net outgoing cooling power during the day (and night). We also present a "Selective+Directional" case where the Selective emitter is combined with the Directional configuration with a broadband reflector to reject the direct-solar radiation. This combined configuration provides the best performance – high maximum cooling power and significantly lower minimum achievable temperature. These results indicate that the directional approach to daytime radiative cooling and its possible combination with current approaches using spectrally selective surfaces could improve passive daytime radiative cooling performance.

Supplementary Note 9: Directional daytime radiative cooling – Applications

The directional approach to passive daytime radiative cooling presented in this work could be useful for applications such as portable refrigeration and concentrated photovoltaic (CPV) cooling. Supplementary Figure 9 shows conceptual drawings of how the directional approach might be implemented to achieve passive cooling in a portable cooler and CPV system in a simple and cost-effective manner.

Supplementary Figure 9a presents one possible embodiment of a portable cooling device that comprises of a band-type reflector that can reflect direct solar radiation. The angular position of the band reflector will depend on the position of the sun in the sky and can be adjusted for a given day. The top surface of the insulated cooler comprises of the mid-IR emitter which has access to the atmosphere and could be covered by a solar-reflecting convection cover which could enable sub-ambient cooling.

Supplementary Figure 9b demonstrates how directional radiative cooling might be implemented in a CPV system. A CPV system typically comprises of dual-axis solar-tracking concentrating optics with the PV cell at its focal point.[5,6] Since the focal axis of the CPV always points towards the sun, direct solar radiation impinging on the back of the PV cell (which does not contribute to electricity generation) can be shaded by a fixed, physically-separated reflecting surface as demonstrated in the present study. The backside of the PV cell should be emitting in mid-IR wavelengths to allow atmospheric radiative cooling and can be solar-reflecting to minimize diffuse-solar absorption to further improve cooling performance. A separate convection cover is not necessary since CPV cells do not require sub-ambient cooling – in fact, convection due to wind should supplement cooling by radiation. Thus it should be possible to achieve radiative cooling in

CPV systems using a directional approach by utilizing the existing solar-tracking configuration and without affecting electricity generation.

Supplementary References

1. Duffie, J. A. & Beckman, W. A. in *Solar Engineering of Thermal Processes* (John Wiley & Sons, Inc., 2013).

2. Raman, A. P., Anoma, M. A., Zhu, L., Rephaeli, E. & Fan, S. Passive radiative cooling below ambient air temperature under direct sunlight. *Nature* **515,** 540–544 (2014).

3. Chen, Z., Zhu, L., Raman, A. & Fan, S. Radiative cooling to deep sub-freezing temperatures through a 24-h day–night cycle. *Nat. Commun.* **7,** 13729 (2016).

4. Zhai, Y. *et al.* Scalable-manufactured randomized glass-polymer hybrid metamaterial for daytime radiative cooling. *Science* **355,** 1062–1066 (2017).

5. Bareis, B. F. Concentrating solar energy receiver. (2004).

6. Verlinden, P. J. *et al.* Performance and reliability of multijunction III-V modules for concentrator dish and central receiver applications. *Conf. Rec. 2006 IEEE 4th World Conf. Photovolt. Energy Conversion, WCPEC-4* **1,** 592–597 (2007).
